# Improving the selenium supply of vegans and omnivores with Brazil nut butter compared to a dietary supplement in a randomized controlled trial

**DOI:** 10.1007/s00394-025-03587-z

**Published:** 2025-02-01

**Authors:** Rebecca Simon, Kristina Lossow, Denny Pellowski, Kristin Kipp, Michaela Achatz, Nicole Klasen, Tanja Schwerdtle, Christine Dawczynski, Anna P. Kipp

**Affiliations:** 1 Department of Nutritional Physiology, Institute of Nutritional Sciences, Friedrich Schiller University Jena, Dornburger Str. 24, 07743 Jena, Germany; 2 TraceAge-DFG Research Unit of Interactions of Essential Trace Elements in Healthy and Diseased Elderly, Potsdam-Berlin-Jena-Wuppertal, Germany; 3 Department of Food Chemistry, Institute of Nutritional Science, University of Potsdam, Potsdam, Germany; 4 Department for Pediatrics, Sophien- and Hufeland Klinikum, Hospital Weimar, Weimar, Germany; 5 Federal Office for Radiation Protection (BfS), Berlin, Germany; 6 German Federal Institute for Risk Assessment (BfR), Berlin, Germany; 7 Junior Research Group Nutritional Concepts, Institute of Nutritional Sciences, Friedrich Schiller University Jena, Jena, Germany

**Keywords:** Veganism, Selenium status, Brazil nut, Selenium supplement

## Abstract

**Purpose:**

A vegan diet is associated with health benefits but may also lead to inadequate intake of essential nutrients. Due to the lower selenium content in plant-based compared to animal-based foods, many vegans do not reach the recommended selenium intake in Europe. The only plant-based food with high selenium content is the Brazil nut, even though there is also a high variability. Therefore, we investigated the effectiveness of Brazil nut butter compared to a dietary supplement as selenium source to improve the selenium status of vegans and omnivores.

**Methods:**

44 vegans and 42 omnivores were randomly assigned to one of three intervention groups, either receiving placebo or consuming additional 55 µg of selenium daily as Brazil nut butter or supplement for two weeks. Serum selenium concentrations, glutathione peroxidase 3 (GPX3), and selenoprotein P (SELENOP) were measured at baseline and after intervention. Additionally, dietary selenium intake was estimated using a five-day dietary protocol.

**Results:**

The estimated selenium intake was significantly lower in vegans compared to omnivores and correlated with all three selenium biomarkers. Independent of the dietary pattern (vegan or omnivore), Brazil nut butter as well as supplement significantly increased serum selenium and SELENOP concentrations, while there were no changes in the placebo groups. Both interventions were equally effective in increasing selenium levels, but the upregulation of SELENOP was more pronounced in vegans than in omnivores.

**Conclusion:**

Brazil nuts are a plant-based source of selenium suitable for vegans and omnivores to improve their selenium status when consumed once in a while.

**Trial registration number and date of registration:**

Clinical trials registration number: NCT05814874, April 18 2023.

**Supplementary Information:**

The online version contains supplementary material available at 10.1007/s00394-025-03587-z.

## Introduction

The vegan diet, the strictest form of vegetarianism, is characterized by an exclusion of all animal-based foods [1]. In recent years, veganism has become increasingly popular in Germany, and the number of people who follow a vegan diet is constantly increasing. In 2008, the National Nutrition Survey II recorded 80,000 vegans [2], and by 2023 the number of vegans had risen to 1.52 million [3]. This group mainly consists of younger people, in particular women with a high level of education [2, 4]. Compared to an omnivorous diet, the vegan diet is characterized by a higher intake of dietary fiber, but a lower overall fat intake, in particular less cholesterol and saturated fatty acids. Micronutrients such as vitamin C, folic acid, and copper are consumed more [5–11]. However, limiting food choices can lead to inadequate intake of some nutrients, such as vitamin D, B_2_, B_12_, calcium, iron, iodine, and zinc [7, 8, 10–12]. Also, selenium might not be sufficiently supplied by a vegan diet [7, 8, 11].

Selenium is an essential micronutrient that is needed for the expression and activity of selenoproteins containing the amino acid selenocysteine. Out of those, glutathione peroxidases (GPX) and thioredoxin reductases are crucial enzymes for maintaining redox homeostasis, while further selenoproteins are important for thyroid hormone synthesis, immune function, and male fertility [13]. In plant-based foods, selenium is bound to different compounds but is mainly present as selenomethionine, whereas in animal-derived foods both selenoamino acids, selenocysteine and selenomethionine, can be found. Supplements usually contain inorganic selenate or selenite [14–16]. All selenocompounds can be converted to selenide, which acts as a precursor for selenoprotein synthesis. In contrast, only selenomethionine can also be incorporated unspecifically into proteins instead of methionine and, as such, might act as selenium storage in the body [17]. Selenium is transported from the liver to organs such as the brain via the transport protein selenoprotein P (SELENOP), which accounts for up to 60% of the selenium in the plasma. In addition, GPX3 is the second extracellular selenoprotein contributing to plasma selenium, which is mainly secreted by the kidneys [18, 19]. Besides serum selenium concentration, GPX3 and SELENOP serve as functional biomarkers for the selenium status [20]. An adequate selenium supply is still difficult to define but may be characterized by serum selenium concentrations in the range of 50–120 μg/L [21]. A concentration of 70–90 μg/L leads to maximal enzyme activity of GPX3, whereas SELENOP only reaches its maximum at a selenium concentration of 100–120 μg/L [22–26]. A plasma selenium concentration of about 120 μg/L appears to protect against some types of cancer [22]. A recent summary of studies from healthy Germans showed that the general population has an average serum selenium concentration of 82 μg/L [27]. In Germany and most other European countries, the selenium supply of humans is mainly ensured through animal foods, as animal feed is usually fortified with selenium in the EU [15, 28]. As plant-based foods grown in Germany only contain low amounts of selenium due to the low selenium content of the soil, they accordingly contribute little to the selenium supply [27]. In comparison, in Germany eggs contain approximately 23 μg Se/100 g, whereas beans only contain up to 6 μg Se/100 g [29]. But consumption of, e.g., imported wheat products from the US provides substantially higher amounts of selenium, as wheat grains from the USA have significantly higher levels of approximately 29 μg/100 g than European wheat with 9 μg/100 g [16, 30]. Therefore, it is very difficult to use standard nutritional survey data to estimate selenium intake because, particularly for plant-based foods, there are large differences in selenium concentrations depending on the geographical region in which they have been grown [15, 27, 28]. Subject to this, it has been described that due to the lower selenium content in a vegan diet compared to an omnivorous diet, many vegans do not reach the daily intake of 70 and 60 µg for men and women, respectively, recommended by the German Nutrition Society [7, 8, 10, 31]. Since serum selenium concentrations reflect dietary selenium intake, vegans demonstrate lower levels compared to individuals following other dietary patterns, indicating a poorer selenium status [6, 32–34]. Accordingly, there is a need to improve the selenium intake in vegans, ideally through the regular consumption of plant-based foods with a high amount of selenium. One plant-based food with a constantly high selenium content is the Brazil nut, which grows exclusively in the Amazon region characterized by a relatively high selenium concentration in the soil [35].

Therefore, we aimed to address the question of whether Brazil nuts could be a useful source of selenium for vegans in comparison to a supplement and whether their effectiveness in improving the selenium status is modulated by the dietary pattern, in this case by a vegan or omnivorous diet. For this purpose, we conducted a randomized, placebo-controlled study to investigate the effects of a daily selenium intervention in the form of a food (Brazil nut butter) or an over-the-counter selenium supplement on serum selenium concentrations and the functional biomarkers GPX3 and SELENOP in people with a vegan or omnivorous diet. As two additional trace elements, zinc and copper serum concentrations were monitored before and after the intervention to study the effects of both interventions on those parameters.

## Subjects and methods

### Study population and study design

A randomized, placebo-controlled study was conducted with apparently healthy volunteers between 18 and 35 years of age who followed either a vegan diet defined as exclusion of all animal products or an omnivorous diet defined as a minimum of weekly consumption of meat and/or sausages with a stable dietary habit for at least one year. Recruitment was carried out via press release, mailing list, social media, and flyers. Subjects were excluded if they followed another diet than the two described ones or changed their diet during the study period, which was assessed by a questionnaire after the intervention period. Individuals who suffered from acute or chronic diseases (e.g., cancer, infection, gastrointestinal diseases, chronic kidney diseases, thyroid diseases, diseases requiring regular phlebotomy), alcoholism or active drug addiction, nut allergy, or were pregnant or breastfeeding were also excluded from the study. In addition, individuals with a baseline serum selenium concentration of > 150 µg/L were not enrolled in the study. All participants gave their informed consent, and the ethics committee of Jena University Hospital (number: 2023-2913_1-BO) approved the protocol. All procedures were in line with the principles of the Declaration of Helsinki. The SelVeg study was registered at Clinical-Trials.gov (Identifier: NCT05814874).

**Fig. 1:**
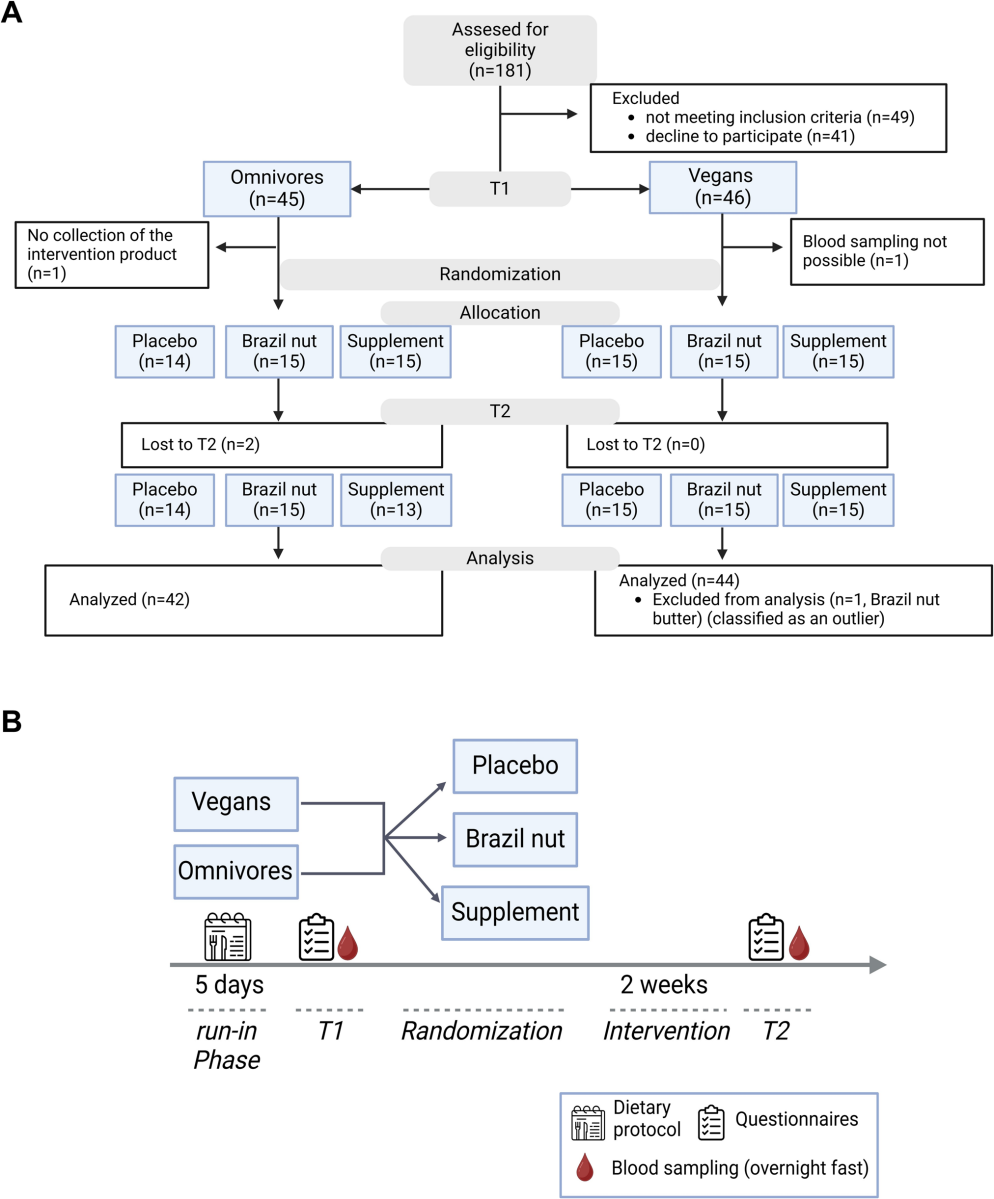
**A** Flow chart and **B** study design. Created in BioRender.com

91 subjects from Jena, Germany, were enrolled (Fig. 1A). After determination of serum selenium levels at baseline, subjects were randomized into one of the three intervention groups with a computer-generated randomization list. Randomization was stratified by dietary pattern (omnivore or vegan). Participants received 55 µg of selenium daily provided by 15 g Brazil nut butter (Naturkostbar AG, Uetendorf, Swiss; macro- and micronutrient content in Tab. S1) or supplement (Mivolis, dm-drogerie markt GmbH + Co. KG, Karlsruhe, Germany) or no additional selenium in case of placebo (vegan capsules (Kapselwelt, Hude, Germany) filled with dextrose) over a period of two weeks (Fig. 1B). The subjects were asked to refrain from consuming additional Brazil nuts or taking selenium supplements during the study phase. In addition, they were instructed to maintain their normal dietary habits during the intervention phase. Compliance was monitored using a checklist, and failure to take the intervention product for more than three days resulted in exclusion from the study. All subjects complied with the intervention regime, but two omnivorous participants did not show up for the second blood drawn and were, therefore, excluded from the study (Fig. 1A). Anthropometric measurements were conducted before and after the intervention period, and the body mass index (BMI) was calculated.

Before and after the intervention, venous blood samples (9 mL) were drawn in the morning after an overnight fast. After coagulation at room temperature, the samples were centrifuged at 2000 × g for 10 min at room temperature, and serum was carefully removed. The serum aliquots were stored at − 80 °C until analysis.

### Dietary assessment

In the non-randomized run-in phase, subjects completed a dietary protocol over five-days (five consecutive days, with one weekend day). For the dietary protocol, the validated template Freiburg food record (*Freiburger Ernährungsprotokoll)* was used, which was provided by PRODI^®^ version 6.4 (Nutri-Science, Stuttgart, Germany) to document the consumed food and drink items and the corresponding portion sizes adapted to German consumption behaviour [36]. Vegan food alternatives, such as vegan drinks, yoghurt, or meat alternatives, were largely included in the food composition database. But new recipes have been created for vegan compound foods such as cakes, cookies, or some side dishes, as these were only available in the food database with animal-based ingredients. The NutriBase^®^ software package calculates daily energy and nutrient intake. However, the database lacks information on the selenium content of food items. Hence, selenium intake was approximated by referencing the food composition database, which provides information on the mineral and vitamin content in different foods offered by the European Food Safety Authority (EFSA) [29]. The FoodEx2 classification used in the database is a standardized system developed by the EFSA that describes and aggregates individual foods hierarchically into food categories. The selenium content of foods is available for seven different EU countries. In order to estimate the selenium intake of our collective, only the available selenium concentrations of foods from Germany were used. Supplement usage was documented separately and factored into the calculation of the estimated selenium intake. Additional other questionnaires were used to obtain information on dietary habits, physical activity, and health status.

### Radioactivity in Brazil nut butter

Radionuclides resulting from the natural decay chains of U-238, U-235, and Th-232, as well as K-40, are ubiquitous in the environment. Plants actively absorb radionuclides together with the nutrients they require for growth. The radionuclide content of the Brazil nut butter was determined using high-purity germanium (HPGe) gamma spectrometry. The principle of gamma spectrometry is that gamma-rays (photons) interact with matter by transmitting their energy to electrons. These excited electrons then lose energy by ionizing and excitation to the detector unit of the gamma spectrometer [37]. 5.43 g of sample material was weighed into a test tube and analysed using a Canberra p-type detector (borehole) with a relative efficiency of 36.6% and a measurement time of 210,000 s. The digital signal was processed using Canberra GmbH DSA 1000 and Genie 2000 was used for activity calculation. K-40 was determined using the gamma line at 1460.83 keV. Ra-228 was determined via its progeny Ac-228 (gamma lines at 338.32 keV, 911.20 keV and 968.97 keV). Pb-212 (238.63 keV) and Tl-208 (583.19 keV and 2614.53 keV) were used to determine Th-228. The gamma line at 46.54 keV was used to determine Pb-210. The gamma lines of Pb-214 (295.22 keV and 351.93 keV) and of Bi-214 (609.31 keV, 1120.29 keV, and 1764.49 keV) were used to analyse Ra-226. U-238 was determined using the gamma line of Th-234 (63.28 keV) [38].

Calculating the dose of public radiation exposure depends on three primary input parameters: the activity concentration of radionuclides in foods, data on food consumption habits, and the effective dose coefficient for ingestion. The annual effective dose by ingestion E (in sieverts) received by an individual in any given year is calculated using the following equation [39]:

$${E}_{\text{Ing},j}=\sum_{n}{U}_{n,j}\cdot \sum_{r}{c}_{n,r }\cdot {g}_{\text{Ing},r,j}$$

${E}_{\text{Ing},j}=$ Annual effective dose by ingestion E [Sv] received by an individual person j.

${U}_{n,j}=$ Annual amount of food item n for average consumption of individual person j [kg].

${c}_{n,r }=$ Mean specific activity of radionuclide r in ingested food item n [Bq/kg].

${g}_{\text{Ing},r,j}=$ Effective dose coefficient for ingestion of radionuclide r for an individual person j [Sv/Bq].

### Analysis of trace element concentrations

The concentrations of trace elements in Brazil nuts (Seeberger GmbH, Ulm, Germany) and Brazil nut butter were determined using inductively coupled plasma-tandem mass spectrometry (ICP-MS/MS). Raw Brazil nuts were subjected to mechanical crushing via mortar milling. Subsequently, Brazil nuts and Brazil nut butter (100–200 mg) underwent microwave-assisted acid digestion (Mars6, CEM, Kamp-Lintfort, Germany). Digested solutions were diluted 1:10 and then submitted to ICP-MS/MS (Agilent ICP-QQQ-MS 8800, Agilent Technologies, Waldbronn, Germany) for quantification of selenium and other minerals, including zinc and copper. Fish muscle (ERM^®^-BB422, European Commission, Joint Research Centre, Geel, Belgium) and pig kidney (ERM^®^-BB186, European Commission, Joint Research Centre, Geel, Belgium) were included as reference materials to ensure method accuracy (approximately 50 mg). External calibration standards for zinc and copper were prepared from a 1000 mg/L stock solution (Carl Roth, Karlsruhe, Germany). Selenium quantification utilized isotope dilution analysis, where an internal standard solution containing 10 µg/L ^77^Se (certified by Trace Sciences International, Ontario, Canada, purchased from Eurisotop SAS, Saarbrücken, Germany) was added. Additionally, germanium (10 µg/L) and rhodium (1 µg/L) (both Carl Roth, Karlsruhe, Germany) were included as internal standards to monitor and correct for signal suppression and matrix effects.

The trace element concentrations in serum were measured by X-ray fluorescence (TXRF) using a benchtop TXRF spectrometer (S4 T-Star^™^, Bruker Nano GmbH, Berlin, Germany) equipped with a molybdenum tube. A gallium solution (1 mg/mL, Thermo Fisher Scientific, Kandel, Germany) was added as internal standard. 10 μL of each sample was placed onto unsiliconized quartz carriers and dried on a heating plate at 40 °C. Analyses were carried out for 750 s in duplicates. Reference ranges indicating normal serum concentrations of selenium, zinc, and copper were defined as 50–120 µg/L, 600–1200 µg/L, and 560–1690 µg/L, respectively [21].

### GPX activity

The GPX activity measurement in serum was conducted via a NADPH-consuming glutathione reductase coupled assay as previously described [34]. Briefly, GPX enzymes catalyse the reduction of hydrogen peroxide (Merck KGaA, Darmstadt, Germany), resulting in the concomitant oxidation of glutathione (Sigma-Aldrich/Merck, Darmstadt, Germany). In a coupled reaction with glutathione reductase (Sigma-Aldrich/Merck) and NADPH (Carl Roth, Karlsruhe, Germany), the oxidized glutathione is converted back to its reduced form. The reduction of NADPH is proportional to GPX activity. Absorbance was measured at 340 nm, with samples diluted by 1:10 and analysed in triplicate. GPX activity was expressed as U/L.

### SELENOP concentration

SELENOP levels were measured using a validated, commercial sandwich ELISA (selenOtest^™^, SelenOmed GmbH, Berlin, Germany). Sample preparation and assay procedures were performed as described by the manufacturer.

### Statistical analysis

The power calculation was based on a previous study, which included 59 healthy subjects assigned to three intervention groups [40]. The increase in serum selenium concentration was used for the power calculation. With a significance level of 5%, a sample size of 15 participants per group resulted in 80% power, ensuring the study’s ability to detect significant effects. The statistical analysis was performed with SPSS (version Premium 29.0.0.). Descriptive statistics were conducted for all parameters and expressed as mean ± standard deviation. Baseline comparisons between the two dietary patterns were conducted using independent Student´s t-test. Comparisons between values at baseline (T1) and after intervention (T2) within each intervention group (placebo, Brazil nut butter, or supplement) were assessed via paired Student's t-test. The effectiveness of the intervention product was evaluated by calculating the percentage change between values at T1 and T2. Two-way ANOVA was performed to assess the effects of dietary pattern, intervention product, and their interaction on the percentage changes, followed by multiple comparisons using Bonferroni or Dunnett-T3 post hoc tests to assess differences between each intervention group. Pearson's coefficient was used to determine correlations between variables. Statistical significance was defined as p < 0.05. Outliers were identified based on the mean difference of the three selenium parameters between T1 and T2. Participants were excluded if differences between the two time points in any of the selenium parameters were greater than 3 times the interquartile range above the third quartile or less than 3 times the interquartile range below the first quartile. One vegan participant was excluded due to a notable decrease in SELENOP level and selenium concentration after the intervention, classifying these values as outliers.

## Results

### Characteristics of subjects at baseline

**Fig. 2:**
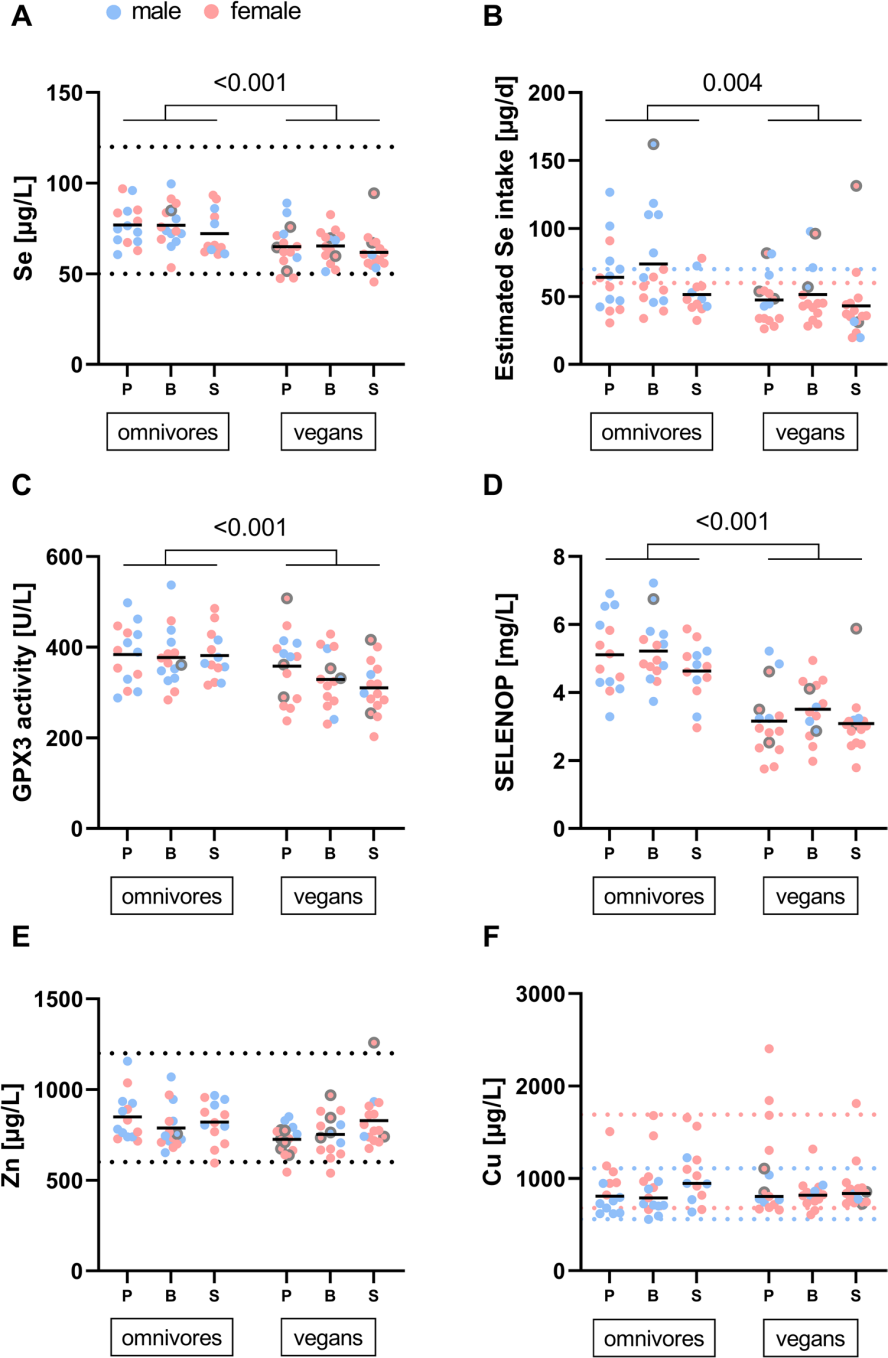
Baseline selenium, zinc, and copper status. **A** Serum selenium concentrations were measured using total reflection X-ray fluorescence spectroscopy (TXRF). **B** The selenium intake was estimated using the EFSA database. **C** Glutathione peroxidase 3 (GPX3) activity was measured by colorimetric assay. **D** Selenoprotein P (SELENOP) levels were measured with ELISA. Serum zinc (**E**), and copper (**F**) concentrations were analysed by TXRF. P = placebo, B = Brazil nut butter, S = supplement. The values are presented by intervention group and separately for omnivores (n = 42) and vegans (n = 44). For one omnivore in the supplement group, the data for the estimated selenium intake is not provided due to a missing dietary protocol. The black line indicates the mean value. Dashed lines indicate reference values (blue shows reference values for men, red for women). The grey border highlights the participants who took supplements containing the respective trace element before the intervention phase. Statistical significance was determined by one-way ANOVA and Bonferroni multiple comparison between intervention groups and unpaired Student's t-test between dietary patterns

The analysis included 44 vegans and 42 omnivores. Participants ages ranged from 19 to 32 years, and their BMI was on average within the normal range (> 19 and < 25 kg/m^2^). In the vegan group, 80% of the participants were female, while in the omnivorous group, both sexes were equally distributed. The general baseline characteristics did not differ between omnivores and vegans, except for BMI and supplement intake (Tab. 1). Vegans showed a significantly lower BMI than omnivores (p = 0.039). Notably, only one vegan participant did not take any supplement, in contrast to 62% of omnivores taking no supplement. Among vegans, the most often used supplements were vitamin B_12_, vitamin D, and multinutrient supplements, while omnivores primarily consumed magnesium, vitamin D, and iron supplements. Twelve vegans and one omnivorous participant took zinc supplements. Copper was supplemented by four vegans, mostly in the form of multinutrient tablets. The concentrations of the trace elements varied per tablet between 2–25 mg and 0.25–2 mg for zinc and copper, respectively. Eight vegans and one omnivorous participant reported using a selenium supplement. The supplements used had different selenium concentrations, ranging between 15–200 μg per tablet and were consumed at different frequencies (daily, monthly, or irregularly). As expected, those vegans showed higher values for the selenium parameters at baseline (Fig. 2A–D).

**Table 1:** Baseline characteristics of omnivores and vegans

	Omnivores (n = 42)	Vegans (n = 44)	Mean differences [95% CI]	p-value
Gender, n (%) male/female	21 (50)/ 21 (50)	9 (20.5)/ 35 (79.5)		
Age (y)^a^	24.83 ± 3.23	24 ± 2.96	0.83 [−0.51, 2.17]	0.221
BMI (kg/m^2^)^a^	24.14 ± 3.71	22.87 ± 2.53	1.61 [0.08, 3.14]	**0.039**
Smoking status, n (%)^b^				1.00
Never	31 (73.8)	33 (75)		
Ex-smoker	6 (14.3)	6 (13.6)		
Current	4 (9.5)	5 (11.4)		
PAL^a^	1.82 ± 0.28	1.83 ± 0.16	− 0.01 [− 0.11, 0.09]	0.872
Supplement, n (%)^b^				** < 0.001**
None	26 (61.9)	1 (2.3)		
1	8 (19)	20 (45.5)		
2	1 (2.4)	12 (27.3)		
> 2	6 (14.3)	11 (25)		
Energy (kcal)^*a*^	2342 ± 610.83	2175 ± 569.03	167.45 [− 87.08, 421.98]	0.194
Carbohydrate (%)^*a*^	42.27 ± 8.21	52.66 ± 5.42	− 10.38 [− 13.37, − 7.40]	** < 0.001**
Dietary fiber (g)^*a*^	24.49 ± 8.11	44.7 ± 14.27	− 20.20 [− 25.18, − 15.22]	** < 0.001**
Protein (%)^*a*^	16.01 ± 3.78	14.34 ± 2.77	1.66 [0.24, 3.1]	**0.024**
Fat (%)^*a*^	37.85 ± 8.27	28.53 ± 5.52	9.33 [6.32, 12.34]	** < 0.001**

Mean ± SD or number (percentage). 95% CI = 95% confidence interval. PAL = Physical activity level. One omnivorous participant did not provide any information about age, smoking status, PAL and supplement intake, and the macronutrient intake could not be calculated due to missing dietary protocol. Statistical significance was determined by ^a^unpaired Student´s t-test or ^b^ Chi-square test/fisher´s exact test. Bold p-values indicate significance

Based on the five-day dietary assessment during the run-in phase, energy, macronutrient, and fiber intake was calculated (Tab. 1). Vegans and omnivores had the same energy intake but differed in their macronutrient and fiber intake. Vegans had a higher carbohydrate (p < 0.001) and fiber (p < 0.001) intake but a lower protein (p = 0.024) and fat (p < 0.001) intake. Within the group of vegans and omnivores, there were no differences between the three intervention groups (placebo, Brazil nut butter, and supplement) for all baseline characteristics except for the smoking status of vegans (p = 0.011). There were more current and ex-smokers among omnivores in the Brazil nut group compared to the omnivorous placebo and supplement groups (Tab. S2).

At baseline, mean serum selenium levels were 76 and 64 μg/L for omnivores and vegans, respectively, and were significantly higher in omnivores compared to vegans (p < 0.001). 7% of vegans had serum selenium concentrations below the reference range of 50–120 μg/L [21], while none of the omnivores were outside this range (Fig. 2A). In line with this, the functional biomarkers GPX3 (Fig. 2C) and SELENOP (Fig. 2D) were significantly lower in vegan than in omnivorous participants (both p < 0.001).

In vegans, the mean estimated dietary selenium intake was 47 μg/day including selenium supplements (41 μg/day without including supplements), while it was 64 μg/day for omnivores (62 μg/day without including supplements) (Fig. 2B). The estimated intake differed significantly between the dietary patterns (p = 0.004). Many participants did not reach the recommended intake of 60 µg/d for women and 70 µg/d for men at the beginning of the SelVeg study. Especially in the vegan group, 84% were below the recommended intake, compared to 63% of omnivores. As expected, a significant correlation was observed between the estimated selenium intake and the selenium concentration at baseline (r = 0.379, p < 0.001). Baseline SELENOP levels and GPX3 activity also correlated significantly with the calculated selenium intake (r = 0.583, p < 0.001 and r = 0.274, p = 0.011). Neither serum zinc nor copper levels differed significantly between the two dietary patterns at baseline (p = 0.066 and p = 0.854). In addition, most participants were within the reference range for both trace elements (Fig. 2E, F). At baseline, there was no difference in any of the trace element status biomarkers between the three intervention groups in omnivores and vegans (Fig. 2A–F).

### Trace element content in Brazil nuts and Brazil nut butter

Prior to the beginning of the study, the trace element concentrations of eight Brazil nuts from five different batches were analysed. In the Brazil nuts, the selenium content ranged from 0.25 to 5.06 µg/g, indicating difficulties to reliably calculate the selenium amount consumed by Brazil nuts. In contrast, the selenium concentration of Brazil nut butter was more homogenous within one batch (3.45–3.65 µg/g). Due to the variation in the selenium content of Brazil nuts, Brazil nut butter was used for the study. The daily consumption of Brazil nut butter was set at 15 g/day in order to supply approximately the same amount of selenium as provided by the supplement, which contained 55 µg in form of sodium selenate. The amounts of zinc and copper in 15 g Brazil nut butter only contributed little to the daily requirement (Table 2).

**Table 2:** Trace element concentrations in Brazil nuts and Brazil nut butter

[µg/g]	Brazil nuts	Brazil nut butter	% of the daily requirement with an intake of 15 g Brazil nut butter
Selenium	1.63 ± 0.47	3.58 ± 0.07	79–92
Zinc	41.20 ± 1.63	43.28 ± 1.09	4–9
Copper	19.42 ± 0.94	17.10 ± 0.3	17–26

Mean ± SD. Brazil nuts n = 8 (from different batches). Brazil nut butter n = 6 (from the same batch). Reference values for nutrient intake according to the German Nutrition Society [41]

Like all foods, Brazil nuts contain natural radionuclides, especially radium [42, 43]. As the result of the gamma spectrometric analysis, the specific activity (Bq/kg) for each radionuclide was calculated (Tab. S3). Consuming 15 g of Brazil nut butter daily for 14 days resulted in an additional radiation exposure of 6.34 µSv/a for each participant of the study (Tab. S4). The natural radiation exposure in the northern hemisphere ranges around 2,400 µSv/a consisting of exposure to cosmic and terrestrial radiation (800–900 µSv/a), inhalation through radon (1,200 µSv/a), and ingestion of food (300 µSv/a) [44, 45]. In relation to this, the additional radiation exposure due to 14 days of consumption is very low. However, a daily consumption of 15 g Brazil nut butter during one year would result in additional radiation exposure of 165 µSv/a. This level of exposure would be relatively high when compared to the average exposure from the ingestion of food of 300 μSv/a.

### Effect of the interventions on the selenium, zinc, and copper status

**Fig. 3:**
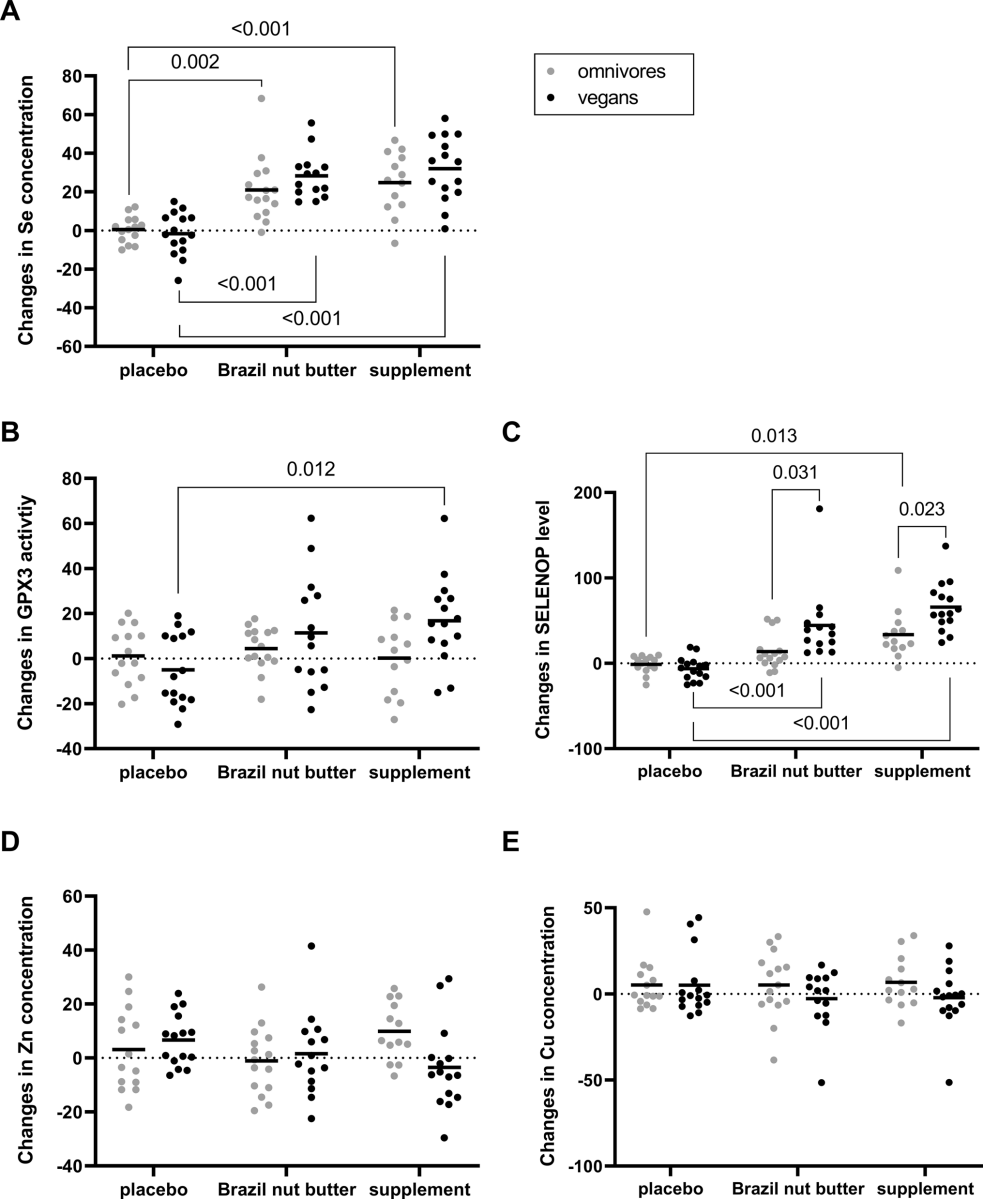
Percentage changes of selenium biomarkers and trace element concentrations by a two-week selenium intervention with Brazil nut butter, supplement, or placebo. Changes in serum selenium concentrations (**A**), glutathione peroxidase 3 activities (GPX3) (**B**), selenoprotein P (SELENOP) levels (**C**), serum zinc (**D**), and copper concentrations (**E**). The black line indicates mean value (n = 13–15). Statistical significance was determined by two-way ANOVA and Bonferroni multiple comparison

The changes of the selenium biomarkers were analysed separately for vegans and omnivores across the three treatment groups (Tab. S5). Following the intervention, serum selenium concentrations increased in both the Brazil nut and supplement groups, but remained unchanged in the placebo groups. In vegans consuming Brazil nut butter, serum selenium levels significantly raised by 18 μg/L (p < 0.001). In the supplement group, serum selenium concentrations also increased by 18.5 μg/L (p < 0.001) but there were no changes in the placebo group (p = 0.349). This effect was mirrored in omnivores, with serum selenium levels significantly increasing during the intervention in both the Brazil nut group (+ 14.7 μg/L, p < 0.001) and the supplement group (+ 16.6 μg/L, p < 0.001), while there was no effect in the placebo group (p = 0.940) (Tab. S5). The results of the two-way ANOVA revealed significant differences in percentage changes in serum selenium levels between the Brazil nut and supplement groups compared to the placebo group, regardless of the dietary pattern. However, there was no significant difference in percentage changes between the Brazil nut and supplement groups themselves (Fig. 3A). Also, the dietary pattern (vegan or omnivore) and the interaction between the intervention product and dietary pattern did not have a significant effect on the changes in serum selenium levels (p = 0.171 and p = 0.330).

In contrast to the selenium concentration, GPX3 activity was only minimally affected by the intervention. Only vegans who consumed the supplement showed a significant increase in activity from 311 U/L to 357 U/L (p = 0.007). No alterations were observed for GPX3 activity of participants consuming Brazil nut butter or placebo, independent of their dietary pattern (Tab. S5). Mean percentage changes in GPX3 activity showed that the intervention products (p = 0.041) as well as the interaction between dietary pattern and intervention product (p = 0.047) influenced GPX3 activity. Vegans consuming the supplement had a significantly higher increase in GPX3 activity than those with placebo (p = 0.012) (Fig. 3B).

SELENOP levels increased in both the supplement groups as well as in the Brazil nut groups. The increase was 1.9 mg/L in vegans (p < 0.001) and 1.4 mg/L in omnivores (p < 0.001) consuming the supplement. In the Brazil nut groups, levels raised by 1.4 mg/L (p < 0.001) in vegans and 0.6 mg/L (p = 0.014) in omnivores. SELENOP levels were stable in omnivores in the placebo group, while in vegans the levels decreased significantly (−0.26 mg/L, p = 0.025) (Tab. S5). One possible explanation for this could be that 8 vegans stopped taking supplements during the study. SELENOP levels were influenced by dietary pattern (p < 0.001), intervention product (p < 0.001) as well as their interaction (p = 0.011). In vegans, both the supplement and Brazil nut butter intervention significantly increased SELENOP compared to the placebo group (p < 0.001). In omnivores, only the supplement group showed a significant difference in SELENOP compared to the placebo group (p = 0.013). In addition, differences between the dietary patterns were observed, with vegans consuming either Brazil nut butter or the supplement having a significantly higher increase compared to their respective group of omnivores (p = 0.031 and p = 0.023) (Fig. 3C).

Copper levels did not change in any of the intervention groups (Tab. S5). Neither the dietary pattern nor the intervention had any influence on the change in copper levels (Fig. 3D). An increase in zinc levels was observed in vegans receiving placebo (p = 0.023) and omnivores taking the supplement (p = 0.006). The other groups showed no changes in zinc levels (Tab. S5). The changes in zinc levels were not influenced by the dietary pattern or the intervention individually, but by the interaction of the two (p = 0.034) (Fig. 3E).

## Discussion

It is well known that Brazil nuts have high selenium concentrations, and accordingly, their consumption increases serum selenium concentrations in humans [40, 46, 47]. However, this is the first study to investigate the selenium status in response to a Brazil nut or supplement intervention while also considering an omnivorous or vegan dietary pattern. In line with the literature, we could show that vegans have a higher risk of poorer selenium status because of the exclusion of animal-derived foods [6, 32–34], which are the main sources of selenium in Europe. Most studies, like ours, reported lower serum selenium concentrations in vegans than in omnivores [6, 32, 34], and also lower SELENOP levels [33]. Results regarding GPX3 activity are inconsistent, with some studies describing lower activities in vegans just like we did [34], while others found no differences between the two dietary patterns [32]. Especially in individuals with an adequate selenium status, GPX3 might not be the most suitable biomarker, as GPX3 activity already saturates at approximately 70–90 μg/L depending on individual variation [25, 26]. As SELENOP only reaches its maximum at higher selenium concentrations around 100–120 μg/L, it covers a wider selenium concentration range than GPX3 [23, 24]. Both biomarkers, SELENOP and GPX3, have the advantage that they reflect the functionally available selenium pool bound to selenoproteins, unlike the measurement of total serum selenium concentration, which includes selenium irrespective of its binding to selenoproteins or other proteins that do not exert selenium-specific functions [48]. However, there are more factors that influence selenium biomarkers. For example, circulating SELENOP is reduced during inflammation [49]. In vitro, it was shown that SELENOP reacts as a negative acute phase protein which is suppressed by cytokines [50]. It is, therefore, suggested to consider all three parameters in parallel when classifying an individual's selenium status [48].

The lower serum selenium concentration and lower activity of selenium-dependent enzymes in vegans reflect the lower selenium intake provided by a vegan diet. A challenge in reliably estimating selenium intake is the variability of selenium concentrations of foods, especially plant-based foods. As the selenium content of soils varies globally, the selenium concentrations of plant-based foods also differ depending on where they were grown [15, 16, 28]. This makes it difficult to accurately determine the selenium intake of individuals based on food frequency questionnaires (FFQ). The food consumption database from the EFSA is the first database that provides selenium concentrations of various food items from different European countries [29]. However, the informative value is also limited here because the import of foods that are grown on selenium-rich soils and, thus, would contribute to the selenium supply is not considered. Therefore, the term ‘estimated selenium intake’ is used, as only an approximate selenium intake can be derived. Subject to this limitation, numerous studies indicate that vegans have a lower selenium intake than omnivores, with selenium intake in vegans often falling below 60 or 70 μg per day recommended by the German Nutrition Society for women and men, respectively [6–8, 10–12, 31]. The estimated selenium intake determined in our study was 62 and 41 μg/day for omnivores and vegans, respectively, with the majority of vegans falling below the recommended intake (84%). Although the use of the estimated selenium intake alone is not sufficient to assess the selenium status, it might further contribute to the information obtained by the three serum biomarkers described. In our study, there was a positive correlation between estimated intake and all three biomarkers, particularly with SELENOP. The combined analysis of selenium biomarkers and the estimated selenium intake in our study reinforces the assumption that vegans show a poorer selenium status than omnivores. This selenium status is still above severe selenium deficiency characterized by serum selenium concentrations below 20 µg/L, which is associated with symptoms contributing to Keshan and Kashin-Beck disease [22, 51]. But even suboptimal selenium levels of around 50 µg/L might have pathophysiological consequences as they have been associated with a compromised immune system [52, 53] and a higher risk of non-communicable diseases such as cardiovascular disease [54–56] and some types of cancer [57–60].

To improve the selenium status, participants received either Brazil nut butter or a selenium supplement as selenium source. While a supplement provides a defined amount of selenium, in this case as selenate, the plant-based food item Brazil nut can be integrated into a normal diet and does not only provide selenium but also other nutrients. Brazil nuts have a characteristic lipid profile with a high content of mono- and polyunsaturated fatty acids and are a valuable source of phytochemicals and minerals [61]. However, regarding their zinc and copper concentration, their contribution to the recommended intake is rather limited, ranging around 10% and 20% for zinc and copper, respectively. We opted for Brazil nut butter due to the lower fluctuations in selenium concentration within the chosen batch and set the daily intake below the recommended intake levels to avoid that the total selenium intake gets close to the tolerable upper intake level (UL) of 255 µg/d [62]. After two weeks with both the Brazil nut butter and the selenium supplement, the serum selenium concentration increased significantly in vegans and omnivores in comparison to the placebo group. The changes over time for the Brazil nut butter groups were 28.3% in vegans and 21% in omnivores. For the supplement group, the increase was 32% and 24.8% in vegans and omnivores, respectively. Studies examining the effect of Brazil nuts on selenium levels in healthy participants also reported a significant increase in selenium levels after Brazil nut consumption [40, 46, 47]. In the study by Thomson et al., plasma selenium levels increased by 64% after consuming two Brazil nuts and by 61% with 100 µg selenomethionine after 12 weeks [40]. This stronger increase could be attributed to both the higher selenium concentration and the longer intervention period. It is well established that the serum selenium concentration increases linearly with higher selenium intake when selenium is provided as selenomethionine [63]. In two studies from Brazil, in which the selenium intake of the subjects was most probably above the UL of 255 µg/day (EFSA) through the consumption of Brazil nuts, selenium concentrations of up to 290 µg/L were reached after the intervention period [46, 47]. The current UL published by the EFSA is based on alopecia as an early adverse effect of excessive selenium exposure [62]. Other authorities, such as the IOM and WHO, set the UL for selenium at 400 µg/day, which considers selenosis as clinical outcome. Chronic selenosis is characterised by symptoms such as brittle, thickened nails, alopecia, garlic odour of the breath, skin lesions, and neurological abnormalities, e.g., fatigue and decreased cognitive function [62]. Thus, our supplementation regime was moderate in terms of total selenium intake (estimated maximum dietary selenium intake of 127 µg/day + 55 µg/day as supplement or Brazil nut butter), and we assured to not over-supplement study participants, e.g., by excluding individuals with initially high serum selenium concentrations.

In contrast to other studies [40, 47], we did not observe a significant increase in GPX3 activity after ingestion of Brazil nut butter or selenium supplement, except for vegans consuming the supplement. Plasma selenium concentrations > 70 µg/L do not result in further increase in GPX3 activity [25]. Since the omnivores already showed baseline serum selenium concentrations > 70 µg/L, it can be assumed that GPX3 had already reached its maximum and that no further increase would have been possible through the intervention. Furthermore, selenium supplementation is supposed to rapidly saturate GPX3 in individuals with lower selenium intake [63]. Herein, we only observed an increase of GPX3 activity in vegans in response to the inorganic supplement, which is quickly available for selenoprotein synthesis. SELENOP levels were increased with Brazil nut butter and the supplement irrespective of the dietary pattern, which fits to the rather low baseline selenium levels where SELENOP has not yet reached a plateau.

Brazil nut butter and selenium supplementation did not significantly differ in their effectiveness in any of the selenium parameters, although a tendency was observed suggesting that the selenium supplement induced stronger effects than the Brazil nut butter. Especially the upregulation of SELENOP was about 20% higher with the supplement in both dietary patterns, and for GPX3 only with the supplement, a significant increase was observed. Selenomethionine, the form of selenium found primarily in Brazil nuts [14], can be non-specifically incorporated into proteins instead of methionine, unlike the inorganic sodium selenate provided by the selenium supplement, which is used exclusively and directly for selenoprotein synthesis [17, 64]. Thus, both selenium sources equally increased the selenium concentration, but the proportion of increase achieved by higher selenoprotein expression appeared to be higher in the supplement group, at least after two weeks of intervention. Overall, due to the lower baseline selenium status of the vegans, they benefited equally from both selenium sources, as SELENOP levels did not differ between the two intervention products. These results indicate that in vegans with a suboptimal selenium status, the selenium form initially has no influence on the effectiveness of improving the selenium status. With higher selenium status as shown here for the omnivores, inorganic selenium appears to be more effective in increasing SELENOP levels, while selenomethionine is entering the methionine pool and shifting the selenium distribution towards retention [65–67]. These differences might be reduced after a longer period of intervention because than the protein turnover would make unspecifically bound selenomethionine available again for the functional selenium pool. As selenomethionine can be further processed in the transsulfuration pathway, several B vitamins, such as vitamin B_12_ and vitamin B_6_, could be limiting factors if they are not sufficiently supplied, which might more often be the case in vegans than in omnivores [5, 68]. In our study, 89% of the vegans supplemented vitamin B_12_, and there was also no sign of reduced vitamin B_6_ intake based on the FFQ data. In rats, unspecific incorporation of selenomethionine instead of methionine into proteins was shown to be influenced by the ratio of both amino acids [69]. This ratio could also be affected by the dietary pattern, as vegans have a lower methionine intake than omnivores [70, 71]. But to address these questions, further intervention studies with different selenium concentrations and intervention time points would be needed to elucidate potential differences between transsulfuration efficiency in vegans and omnivores.

It should be noted that the study has some limitations. First of all, the sample size was relatively small. Although a power analysis has been carried out, more participants would have been desirable, particularly for the subgroup analysis. Second, in contrast to the omnivores, the gender distribution in the vegan group was not balanced. Therefore, gender-specific differences could not be addressed and may have biased the results even though previous studies do not indicate sex differences for the parameters analysed herein [32, 72]. Furthermore, no conclusions can be drawn as to whether the intervention had any influence on dietary habits, as nutrient intake was only recorded at the beginning of the study even though participants were instructed to maintain their normal dietary habits during the intervention phase. This could be particularly relevant in the Brazil nut group, as Brazil nut butter consumption could lead to changes in the intake of other foods.

In summary, Brazil nuts clearly have the potential to improve the selenium status in individuals with low selenium supply, thus providing vegans with a plant-based option for enhancing their selenium status. In contrast to the selenium supplement, Brazil nuts provide further valuable nutrients such as polyunsaturated fatty acids and minerals in addition to selenium. However, we could not show any effects of Brazil nut consumption on serum zinc or copper concentrations, indicating that their contribution to the daily zinc and copper intake is negligible. Furthermore, regular Brazil nut consumption is more expensive than taking a standard over-the-counter supplement (e.g., in the SelVeg study, Brazil nut butter cost 1.12 € per day in contrast to 0.05 €/day for the supplement). Overall, an unrestricted recommendation for regular Brazil nut consumption remains challenging due to i) the large variation in selenium concentrations of the nuts resulting in potentially harmful effects due to excessive selenium intake. Even though the variation of selenium concentrations can be reduced by using Brazil nut butter instead of single nuts, there will most probably also be differences between batches and distributors regarding the selenium content of Brazil nut butter. ii) Brazil nuts have a relevant amount of natural radionuclides [42, 43]. The effective dose related to the consumption of 15 g of Brazil nut butter for 14 days each was relatively low. Thus, while Brazil nuts serve as a temporary option for boosting the selenium status, they may not be suitable for a regular supplementation over a lifelong period.

## Supplementary Information

Below is the link to the electronic supplementary material.

Supplementary file1 (DOCX 34 KB)

## Data Availability

The raw data that support the findings of this study are not openly available due to reasons of sensitivity and are available from the corresponding author upon reasonable request.
