# Supplementary material for: Improving the selenium supply of vegans and omnivores with Brazil nut butter compared to a dietary supplement in a randomized controlled trial
Source: Eur J Nutr. 2025 Feb 1;64(2):74. doi: 10.1007/s00394-025-03587-z (PMC11787226; doi:10.1007/s00394-025-03587-z)
Supplement: Supplementary file 1 — Supplementary file1 (DOCX 34 KB) [file 394_2025_3587_MOESM1_ESM.docx]

***Improving the selenium supply of vegans and omnivores with Brazil nut butter compared to a dietary supplement in a randomized controlled trial***

***Rebecca Simon^1^, Kristina Lossow^1,2^, Denny Pellowski^2,3^,*** ***Kristin Kipp^4^, Michaela Achatz^5^, Nicole Klasen^5^, Tanja Schwerdtle^2,3,6^, Christine Dawczynski^7^, Anna P. Kipp^1,2^***

**Supplementary Information**

**Table S1.** Macro- and micronutrient content of Brazil nut butter

| Pro 100 g Brazil nut butter |  |
| --- | --- |
| Fat | 66 g |
| of which saturated fatty acids | 15.1 g |
| of which unsaturated fatty acids | 24.6 g |
| of which polyunsaturated fatty acids | 20.6 g |
| Carbohydrates | 12 g |
| of which sugar | 2 g |
| Dietary fiber | 8 g |
| Protein | 14 g |
| Thiamine | 0.6 mg |
| Vitamin E | 5.7 mg |
| Calcium | 160 mg |
| Iron | 2.4 mg |
| Potassium | 659 mg |
| Copper | 1.7 mg |
| Magnesium | 376 mg |
| Manganese | 1.2 mg |
| Phosphorus | 725 mg |
| Selenium | 1917 µg |
| Zinc | 4.1 mg |

*According to the food label of the manufacturer Naturkostbar AG.*

**Table S2.** Baseline characteristics of participants divided by intervention groups

|  | Omnivores | | | | Vegans | | | |
| --- | --- | --- | --- | --- | --- | --- | --- | --- |
|  | Placebo  (n=14) | Brazil nut  (n=15) | Supple-ment  (n=13) | p-value | Placebo  (n=15) | Brazil nut  (n=14) | Supple-ment  (n=15) | p-value |
| Gender, n (%) male/female | 8 (57.1)/  6 (42.9) | 8 (53.3)/  7 (46.7) | 5 (38.5)/  8 (61.5) |  | 4 (26.7)/  11 (73.3) | 3 (21.4)/  11 (78.6) | 2 (13.3)/  13 (86.7) |  |
| Age (y)^a^ | 24.86 ± 3.23 | 25.60 ± 3.27 | 23.83 ± 3.19 | 0.379 | 23.67 ± 3.44 | 24.64 ± 3.25 | 23.73 ± 2.19 | 0.655 |
| BMI (kg/m^2^)^a^ | 24.06 ± 3.63 | 24.65 ± 3.84 | 24.75 ± 5.6 | 0.907 | 22.35 ± 2.71 | 23.70 ± 3.19 | 22.62 ± 1.33 | 0.450 |
| Smoking status, n (%)^b^ |  |  |  | **0.011** |  |  |  | 0.119 |
| Never | 13 (92.9) | 7 (46.7) | 11 (84.6) |  | 9 (60.0) | 11 (78.6) | 13 (86.7) |  |
| Ex-smoker | 1 (7.1) | 5 (33.3) | 0 |  | 3 (20.0) | 3 (21.4) | 0 |  |
| Current | 0 | 3 (20.0) | 1 (7.7) |  | 3 (20.0) | 0 | 2 (13.3) |  |
| PAL^a^ | 1.79 ± 0.22 | 1.89 ± 0.37 | 1.77 ± 0.19 | 0.479 | 1.81 ± 0.12 | 1.86 ± 0.20 | 1.81 ±  0.16 | 0.677 |
| Supplement, n (%)^b^ |  |  |  | 0.217 |  |  |  | 0.818 |
| None | 8 (57.1) | 9 (60.0) | 9 (69.2) |  | 0 | 0 | 1 (6.7) |  |
| 1 | 5 (35.7) | 3 (20.0) | 0 |  | 5 (33.3) | 8 (57.1) | 7 (46.7) |  |
| 2 | 0 | 1 (6.7) | 0 |  | 5 (33.3) | 3 (21.4) | 4 (26.7) |  |
| >2 | 1 (7.1) | 2 (13.3) | 3 (23.1) |  | 5 (33.3) | 3 (21.4) | 3 (20.0) |  |
| Energy (kcal)*^a^* | 2425 ± 696.52 | 2543 ± 458.5 | 1998 ± 568. | 0.054 | 2486 ± 754.55 | 2045 ± 407.55 | 1987 ± 335.85 | 0.088 |
| Carbohydrate (%)*^a^* | 40.97 ± 11.59 | 42.59 ± 6.44 | 43.39 ± 5.48 | 0.752 | 54.28 ± 4.77 | 52.88 ± 4.41 | 50.82 ± 6.54 | 0.215 |
| Dietary fiber (g)*^a^* | 26.78 ± 8.74 | 25.39 ± 8.93 | 20.71 ± 4.87 | 0.142 | 49.97 ± 17.64 | 43.84 ± 12.59 | 40.22 ± 10.72 | 0.209 |
| Protein (%)*^a^* | 16.33 ± 4.51 | 15.98 ± 4.05 | 15.66 ± 2.59 | 0.907 | 13.24 ± 2.06 | 15.16 ± 3.01 | 14.68 ± 2.97 | 0.149 |
| Fat (%)*^a^* | 39.71 ± 10.75 | 36.51 ± 7.64 | 37.37 ± 5.53 | 0.575 | 27.53 ± 5.03 | 28 ± 4.52 | 30.02 ± 6.74 | 0.433 |

*Mean ± SD or number (percentage).* *PAL = Physical activity level. One omnivorous participant (supplement group) did not provide any information about age, smoking status, PAL, and supplement intake. Also, the macronutrient intake could not be calculated due to missing dietary protocol. Statistical significance was determined by ^a^ one-way ANOVA and Bonferroni multiple comparison, or ^b^ Chi-square test/fisher´s exact test. Bold p-values indicate significance.*

**Table S3.** Specific activity of natural radionuclides in Brazil nut butter

| Radionuclide | Specific activity in Bq/kg | |
| --- | --- | --- |
| K-40 | | 180 ± 20 |
| Ra-228 | | 29 ± 3 |
| Th-228 | | 21 ± 3 |
| Pb-210 | | < 5 |
| Ra-226 | | 27 ±3 |
| U-238 | | < 5 |
|  | |  |

**Table S4.** Model calculations of the annual effective dose for adults. Three exposure scenarios were calculated for adults. The scenarios were based on the assumption that the individual person j would consume (i) 15 g of Brazil nut butter daily over a period of 14 days or (ii) 15 g of Brazil nut butter daily over a period of 365 days.

| Consumption per year in [kg/a] | Radionuclide | Effective dose coefficient for ingestion^3^ in Sv/Bq | Mean specific activity in Bq/kg | Effective dose in μSv/a |
| --- | --- | --- | --- | --- |
| 0.21^1^ | K-40 | 6.20*E-09 | 180 | 0.23 |
|  | Ra-228 | 6.90*E-07 | 29 | 4.20 |
|  | Th-228 | 7.20*E-08 | 21 | 0.32 |
|  | Ra-226 | 2.80*E-07 | 27 | 1.59 |
| **Sum** | | **6.34** | | |
| 5.48^2^ | K-40 | 6.20*E-09 | 180 | 6.11 |
|  | Ra-228 | 6.90*E-07 | 29 | 110.00 |
|  | Th-228 | 7.20*E-08 | 21 | 8.28 |
|  | Ra-226 | 2.80*E-07 | 27 | 41.40 |
| **Sum** | | **165.00** | | |
| *1: This corresponds to 15 g daily for 14 days*  *2: This corresponds to 15 g daily for 1 year*  *3: Bundesministerium der Justiz (Hrsg.) (2001). Dosiskoeffizienten bei äußerer und innerer Strahlenexposition. Bekanntmachung im Bundesanzeiger am 28. August 2001, Beilage Nr. 160 a und b* | | | | |

**Table S5.** Effect of Brazil nut butter, selenium supplement, and placebo on absolute values for selenium biomarkers and trace elements

|  | before | | | | after | | | | Mean differences  [95 % CI] | | | | | | p-values | | Change [%] |
| --- | --- | --- | --- | --- | --- | --- | --- | --- | --- | --- | --- | --- | --- | --- | --- | --- | --- |
| Serum Se (μg/L) | |  | | | |  | | | |  | |  | |  |  |  |  |
| Placebo | 76.89 ± 11.33 | | | | 77.00 ± 10.28 | | | | 0.11 [-2.96, 3.18] | | | | | | 0.940 | | 0.52 |
| Brazil nut | 76.76 ± 11.63 | | | | 91.44 ± 7.92 | | | | 14.68 [9.51, 19.84] | | | | | | **<0.001** | | 20.98 |
| Supplement | 72.11 ± 12.11 | | | | 88.72 ± 10.18 | | | | 16.61 [10.46, 22.77] | | | | | | **<0.001** | | 24.79 |
| Placebo | 64.99 ± 12.08 | | | | 63.27 ± 10.12 | | | | -1.72 [-5.53, 2.09] | | | | | | 0.349 | | -1.64 |
| Brazil nut | 65.39 ± 8.94 | | | | 83.42 ± 10.30 | | | | 18.03 [14.21, 21.86] | | | | | | **<0.001** | | 28.28 |
| Supplement | 61.75 ± 11 | | | | 80.24 ± 8.8 | | | | 18.49 [13.41, 23.56] | | | | | | **<0.001** | | 31.94 |
| GPX3 activity (U/L) | |  | | | |  | | | |  | |  | |  |  |  |  |
| Placebo | 383.95 ± 65.84 | | | | 383.54 ± 54.22 | | | | -0.41 [-30.01, 29.19] | | | | | | 0.977 | | 1.18 |
| Brazil nut | 377.46 ± 64.44 | | | | 392.37 ± 66.48 | | | | 14.92 [-5.99, 35.83] | | | | | | 0.148 | | 4.4 |
| Supplement | 381.75 ± 53.85 | | | | 380.04 ± 71.4 | | | | -1.71 [-39.78, 36.36] | | | | | | 0.924 | | 0.19 |
| Placebo | 358.13 ± 75.67 | | | | 333.19 ± 56.01 | | | | -24.94 [-57.07, 7.18] | | | | | | 0.118 | | -5.01 |
| Brazil nut | 328.57 ± 62.83 | | | | 356.17 ± 60.4 | | | | 27.61 [-15.27, 70.48] | | | | | | 0.188 | | 11.38 |
| Supplement | 310.51 ± 58.34 | | | | 356.94 ± 62.69 | | | | 46.43 [14.65, 78.22] | | | | | | **0.007** | | 16.8 |
| SELENOP (mg/L) | |  | | | |  | | | |  | |  | |  |  |  |  |
| Placebo | 5.11 ± 1.11 | | | | 5.03 ± 1.16 | | | | -0.08 [-0.39, 0.23] | | | | | | 0.591 | | -1.26 |
| Brazil nut | 5.22 ± 0.91 | | | | 5.84 ± 0.94 | | | | 0.62 [0.14, 1.09] | | | | | | **0.014** | | 13.61 |
| Supplement | 4.63 ± 0.84 | | | | 6.01 ± 0.78 | | | | 1.38 [0.84, 1.93] | | | | | | **<0.001** | | 33.5 |
| Placebo | 3.16 ± 1.04 | | | | 2.9 ± 0.89 | | | | -0.26 [-0.48, -0.04] | | | | | | **0.025** | | -6.42 |
| Brazil nut | 3.5 ± 0.84 | | | | 4.86 ± 0.93 | | | | 1.36 [0.88 ,1.84] | | | | | | **<0.001** | | 44.48 |
| Supplement | 3.09 ± 0.88 | | | | 4.98 ± 1.01 | | | | 1.9 [1.55, 2.25] | | | | | | **<0.001** | | 65.88 |
| Serum Zn (μg/L) |  | |  |  |  | |  |  | | |  | |  | | |  |  |
| Placebo | 849.06 ± 130.57 | | | | 862.77 ± 92.14 | | | | 13.71 [-58.04, 85.45] | | | | | | 0.687 | | 3.14 |
| Brazil nut | 787.94 ± 119.42 | | | | 773.38 ± 108.37 | | | | -14.56 [-68.92, 39.81] | | | | | | 0.575 | | -1.05 |
| Supplement | 820.71 ± 116.27 | | | | 899.05 ± 138.00 | | | | 78.33 [27.38, 129.29] | | | | | | **0.006** | | 9.9 |
| Placebo | 724.92 ± 81.42 | | | | 767.92 ± 61.92 | | | | 43 [6.78, 79.22] | | | | | | **0.023** | | 6.67 |
| Brazil nut | 752.62 ± 119.02 | | | | 753.74 ± 99.15 | | | | 1.12 [-63.26, 65.5] | | | | | | 0.971 | | 1.59 |
| Supplement | 828.72 ± 144.9 | | | | 785.27 ± 88.52 | | | | -43.44 [-121.99, 35.10] | | | | | | 0.255 | | -3.49 |

Continued **Table S5**

| Serum Cu (μg/L) |  |  | |  | |  | |  |  |  |
| --- | --- | --- | --- | --- | --- | --- | --- | --- | --- | --- |
| Placebo | 871.65 ± 247.92 | | 910.70 ± 270.38 | | 39.05 [-23.69, 101.79] |  | 0.202 | 3.14 | |  |
| Brazil nut | 888.29 ± 312.38 | | 910.39 ± 305.95 | | 22.11 [-109.03, 153.25] |  | 0.723 | -1.05 | |  |
| Supplement | 1035.93 ± 313.36 | | 1088.09 ± 298.98 | | 52.17 [-29.93, 134.26] |  | 0.191 | 9.9 | |  |
| Placebo | 1069.79 ± 522.33 | | 1096.66 ± 469.64 | | 26.87 [-70.76, 124.51] |  | 0.564 | 6.67 | |  |
| Brazil nut | 838.51 ± 166.85 | | 793.01 ± 99.58 | | -45.49 [-160.41, 69.45] |  | 0.408 | -2.72 | |  |
| Supplement | 909.84 ± 275.78 | | 855.05 ± 132.00 | | -54.79 [-198.13, 88.55] |  | 0.426 | -2.12 | |  |

*Mean* ± *SD. Light grey indicates values for omnivorous groups and darker grey for vegan groups (n = 13-15). 95 % CI = 95 % confidence interval. % changes = Percentage changes. P-values for changes between baseline and after intervention. Statistical significance was determined by paired Student´s t-test. Bold p-values indicate significance.*
